# A Case of Suppuration of the Brain

**Published:** 1858-09

**Authors:** Wm. H. Philpot

**Affiliations:** Talbot County, Ga.


### ARTICLE II.

*A Case of Suppuration of the Brain.* By Wm. H. Philpot, M. D., of Talbot County, Ga.

Dafny, a negro woman some thirty-five or forty years old, belonging to the estate of Mrs. Crawford, in possession of T. R. Lumsden, Esq., of this county, had been sick for several days. I was called to see her Sunday evening the 20th June last.

She complained of some pain in the region of the lumbar vertebræ, and of a dull heavy feeling in right arm, and of being unable to move it. Suffered no pain in the head at that time, but was subject to the headache frequently. Pulse some 90 beats and regular, tongue a little furred. On using pressure upon the Dorsal spine, she complained of pain in the epigastric region, and said the pain would ascend or descend in front, when pressed up and down the spine. She was very sensitive to the touch, and complained of being sore all over.

She had been confined within a few days of two months ago, her labor was not difficult, nor as far as she could judge any way unusual. Her Lochia continued the length of time it was usual with her. She was moved by Railroad and wagon from Fort Valley, in this State, to this County, when her baby was some 3 or 4 weeks old, and had done but a few days work in the farm siuce confinement, previous to her attack. She was very subject to cephalalgia, and when very painful, it was attended by spasmodic action. I could ascertain nothing of her past history, except from herself, and was inclined to the opinion, that here we had a case of hysterics, and prescribed a brisk cathartic, and a mixture of Valerian and Assafœtida, and promised to see her next morning.

On calling, she said she was no better, and had the headache, could move to some extent her arm and leg, both of which she complained of feeling dead, but would cry out lustily upon being pinched upon either. Her pulse was slow and full, and she seemed dull and slow, very slow in speech, presenting a haggard appearance, manifesting a loss more or less of mental action, and indicating pressure either from coagula of blood or effusions of some kind upon the brain. She would persist in the idea she could not move either her right arm or leg, and yet when pinched would quickly move them. I was inclined, as was her master, to believe her somewhat deceitful.

I bled her largely from the arm and from cups to the back of her neck, used counter irritation to the spine, with a stream of cold water frequently during the day to her head, and the application of an electro magnetic battery. Her bowels had acted but slightly, and gave some pills of Croton Tiglium. For the next two days she grew better and again worse frequently, and when hurriedly called about noon on the 23d, I found her in a comatose and insensible condition, could be roused with difficulty, some dysphagia and great dyspnea. I bled her again, and applied cold water with counter irritation to her extremities.

I had my friend Dr. W. P. Mathews, of Prattsburg, called for consultation. We continued the water, the battery, and counter irritation, and gave her internally a mixture of valerian and turpentine.

The poor girl soon lost the power of deglutition, lying in this insensible and comatose condition, with spasms of the muscles of the neck and face on the right side for some forty-eight hours and died on the evening of the 26th.

Dr. Mathews being with me, we made a post mortem examination. On opening the cranium, the membranes were found unusually vascular, containing serum within them. Upon removing them, we found an abscess near the center of the right hemisphere, the same side her paralysis existed. The abscess contained some considerable amount of pus, the walls of the cavity being formed by the surrounding brain, and its membranes. Upon inserting my finger as far as it could reach, the walls of the cavity in the mass of the brain felt rough and very uneven. The blood vessels in left hemisphere seemed engorged with blood.

We opened the thorax, and found old adhesions of both lungs, they presenting an unhealthy appearance, could detect no disease of the heart. Did not examine the cavity of the abdomen.

Inflammation of the substance of the brain may be chronic, and some of the premonitory symptoms may be present for a long time. " May exist even for weeks, months, a year or a longer period," and the progress of the disease be slow, marked by occasional affections of the head, and symptoms of general irritation. It may prove fatal in this stage or from its consequences, when we have infiltration of the brain either with blood, pus or serum. That Dafny labored under cerebritis, I am led to believe from her own statement, suffering as she did for a long time with occasional "pain in the head, it feeling hot, heavy, and full, with sometimes jerking spasms." This disease is not attended in its course with any general reaction, or with frequently any striking symptoms of disturbed health, until the patient is seized with coma and insensibility, and sinks from exhaustion. Affecting as it does so delicate a structure, having no outlet for effused fluids, and life itself being so dependent on its proper functions, the disease must be considered always attended with great danger, the suppuration frequently taking place very rapidly, having been known to form in the short space of twenty-four hours. In Dafny's case the paralysis existed on the right side, and the abscess was found in the right hemisphere. Here was an anomaly, and all pathologists inform us, one difficult to account for, because the decussation of the fibres of the medula oblongata should, and as a general law, does cause the paralysis to occur on the side opposite to that in which the extravasation is seated.

